# Microbial metagenomic approach uncovers the first rabbit haemorrhagic disease virus genome in Sub-Saharan Africa

**DOI:** 10.1038/s41598-021-91961-2

**Published:** 2021-07-01

**Authors:** Anise N. Happi, Olusola A. Ogunsanya, Judith U. Oguzie, Paul E. Oluniyi, Alhaji S. Olono, Jonathan L. Heeney, Christian T. Happi

**Affiliations:** 1 Department of Veterinary Pathology, Faculty of Veterinary Medicine, University of Ibadan, Ibadan, Nigeria; 2 Department of Biological Sciences, Faculty of Natural Sciences, Redeemer’s University, Ede, Nigeria; 3 African Centre of Excellence for Genomics of Infectious Diseases (ACEGID), Redeemer’s University, Ede, Osun State Nigeria; 4 Lab of Viral Zoonotics, Department of Veterinary Medicine, University of Cambridge, Cambridge, UK

**Keywords:** Evolution, Genetics, Microbiology, Molecular biology

## Abstract

Rabbit Haemorrhagic Disease (RHD) causes high morbidity and mortality in rabbits and hares. Here, we report the first genomic characterization of lagovirus GI.2 virus in domestic rabbits from sub-Saharan Africa. We used an unbiased microbial metagenomic Next Generation Sequencing (mNGS) approach to diagnose the pathogen causing the suspected outbreak of RHD in Ibadan, Nigeria. The liver, spleen, and lung samples of five rabbits from an outbreak in 2 farms were analyzed. The mNGS revealed one full and two partial RHDV2 genomes on both farms. Phylogenetic analysis showed close clustering with RHDV2 lineages from Europe (98.6% similarity with RHDV2 in the Netherlands, and 99.1 to 100% identity with RHDV2 in Germany), suggesting potential importation. Subsequently, all the samples were confirmed by RHDV virus-specific RT-PCR targeting the VP60 gene with the expected band size of 398 bp for the five rabbits sampled. Our findings highlight the need for increased genomic surveillance of RHDV2 to track its origin, understand its diversity and to inform public health policy in Nigeria, and Sub-Saharan Africa.

## Introduction

Rabbit haemorrhagic disease (RHD) is a highly infectious and deadly viral haemorrhagic disease of rabbits. The disease is caused by Rabbit haemorrhagic disease virus (RHDV) a *Lagovirus* of the *Caliciviridae* family^1^. Within the lagovirus, RHDV is classified as GI genogroup. Members of this genogroup include the GI.1, GI.2, GI.3 and GI.4 genotypes. The GI.1 is the former G1 to G6 groups and the GI.2 is the earlier classified RHDV2/b. GI.1is divided into variants GIl.1a to Gl.1d^2^.

The RHDV genome is a positive sense and single stranded RNA of approximately 7437 nucleotides in length^1^. There are two open reading frames (ORFs); ORF1 encoding the seven nonstructural proteins (RdRp, RNA-dependent RNA polymerase, p16, p23, p29, helicase, VPg and protease) and the major capsid structural proteins VP1/VP60) and ORF2 encoding the minor structural proteins VP2/VP1^3–5^.

The virus causes a high morbidity and mortality rates, killing more than 90% of infected adult animals in 2–3 days following infection^6,7^. This disease causes economic losses to the rabbit meat and fur industry and great negative ecological impact in wild rabbit population^7,8^. RHD is among the diseases notifiable to the World Organization for Animal Health (OIE).

Transmission of the virus is via nasal, oral, conjunctival routes with mechanical transmission by insects or fomites^9^. The virus is also shed through excretions from infected animals^10^. The lesions of RHD are usually due to circulatory and degenerative disorders. The primary lesions include hepatic necrosis and petechial haemorrhages in multiple organs. However, the most severe form of these lesions appears in the liver, trachea, and lungs^11^. The virus also promotes fatal hepatitis in adult rabbits^12^.

Rabbit haemorrhagic disease was first reported in China in 1984^13^ where over 140 million rabbits were killed in the course of an outbreak^14^. The subsequent spread of the virus was reported in Europe and other continents^15–21^. This disease has been reported in some African countries like Tunisia, Republic of Benin, Morocco, Cape Verde and Egypt^11,22–24^.

A new GI.2 genotype of RHDV was later discovered in France in 2010^11^ and is now reported globally^26–33^. GI.2 was responsible for major outbreaks causing deaths in previously vaccinated adult rabbits as well as young rabbits known to be resistant to disease induced by classical RHD^20,25,31,34^. In sub-Saharan Africa, outbreaks of GI.2 have been confirmed by enzyme immunoassays (EIA) in domestic rabbits from Republic of Benin^23^ and PCR from Cote d’Ivoire^31^. However, to date there are no available virus genome data from any Sub-Saharan African country, rendering it difficult to trace their origin, evolution, and genetic diversity.

Following devastating outbreaks of suspected cases of RHD affecting several rabbitries from the southwestern region of Nigeria in the first few months of the year 2020, there were farms with no surviving rabbits and some sustained considerable economic losses. In August 2020, two smallholding farms (Farm A and Farm B) reported high mortalities. The two farms were visited for investigations, post-mortem examinations, and sample collections for molecular diagnosis. During the outbreak, farmers had observed symptoms similar to those of RHD. Exotic breeds of rabbits of all ages and both sexes were fatally affected. The symptoms reported by the farmers were anorexia (a day prior to death), clear mucoid lacrimal discharge, lethargy, bleeding from the oral and nasal orifices and sudden death. Five carcasses from both farms were examined grossly and samples collected for diagnosis. Here we report the use of microbial metagenomics sequencing to uncover the first genomic characterization and whole genome of the GI.2 in sub-Saharan Africa from an outbreak of RHD in Nigeria.

## Results

### Gross post mortem findings

The carcasses were in good body condition but slightly autolyzed (5/5, that is five out of 5 carcasses). The oral and ocular mucous membranes were mildly pale (5/5). There were a few multifocal widespread petechial haemorrhages on the ventral and dorsal abdominal subcutaneous muscle (2/5), as well a few multifocal petechial haemorrhages spread on the pleura surface of the lungs (2/5). A focal pinpoint haemorrhage was also observed on the kidney (1/5), heart (1/5), liver (1/5) and on the adventitia surface of the trachea (2/5). There was ballooning of the large intestine (1/5) and mild accentuation of the lobular pattern of the liver (1/5).

### RT-PCR

We conducted two sets of RT-PCRs using different primers sets. In the first RT-PCR (RT-PCR1), two samples (RT2 and RT4) out of five were positive for RHDV targeting the VP60 gene (Table 1). Bands were confirmed on 1% agarose gel electrophoresis at regions between 1500 and 2000 bp (results not shown).

**Table 1:** Rabbit sample demographics with molecular results.

Sample ID	Sex	Age	RT-PCR1	RT-PCR2	Genome sequence
RT1	Female	8 weeks	Negative	Positive	No
RT2	Male	8 weeks	Positive	Positive	Yes (partial)
RT3	Male	5 weeks	Negative	Positive	No
RT4	Male	3 weeks	Positive	Positive	Yes (partial)
RT5	Male	Adult	Negative	Positive	Yes (full)

**Figure 1:**
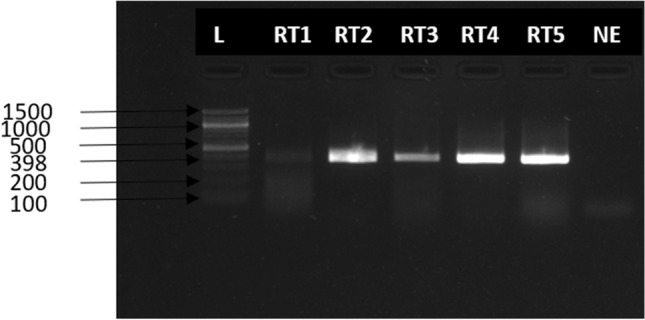
Agarose gel electrophoresis (1% agarose) results of RT-PCR amplified products using specific primers that target the VP60 gene of RHDV. L = DNA maker (5000 bp), RT1 = Rabbit tissue sample 1, RT2 = Rabbit tissue sample 2, RT3 = Rabbit tissue sample 3, RT4 = Rabbit tissue sample 4, RT5 = Rabbit tissue sample 5, *NE* negative extract.

Following sequencing, a second RT-PCR (RT-PCR2) was conducted and all five samples (RT1-RT5) were positive for RHDV targeting the 398 bp region of the VP60 (capsid) gene (Table 1) of the lagoviruses. Bands were confirmed on 2% agarose gel electrophoresis at 398 bp regions (Fig. 1). These samples were confirmed to be RHDV2 by sequencing and bioinformatic analysis.

### Sequencing

**Figure 2:**
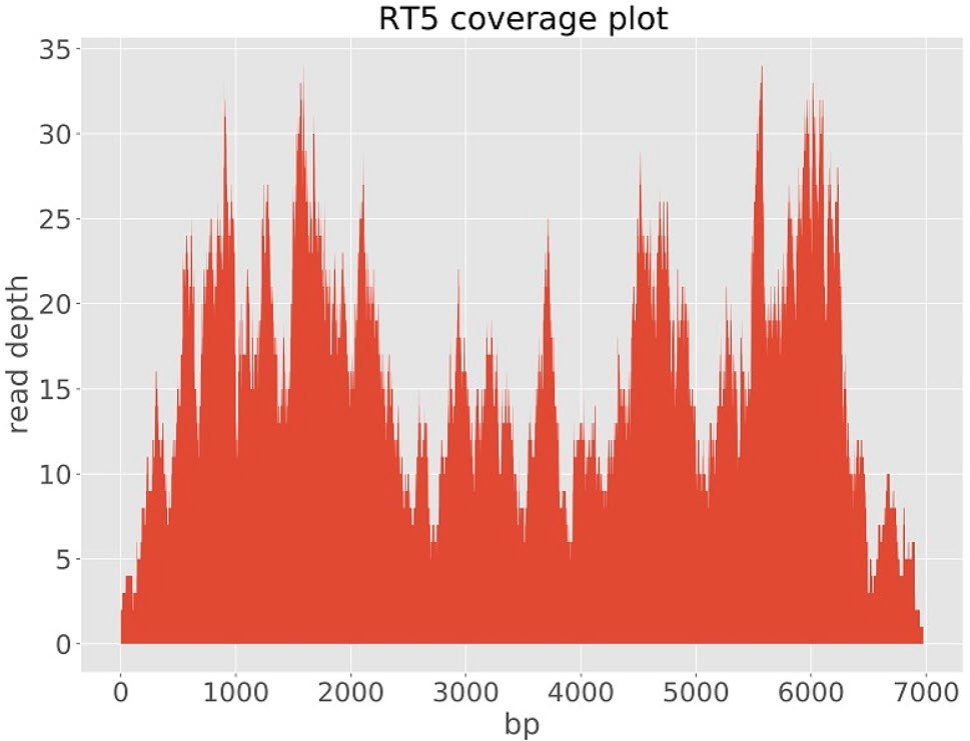
lllumina read coverage across Rabbit Hemorrhagic Disease Virus (GI genogroup) genome assembly from sample RT5.

**Figure 3:**
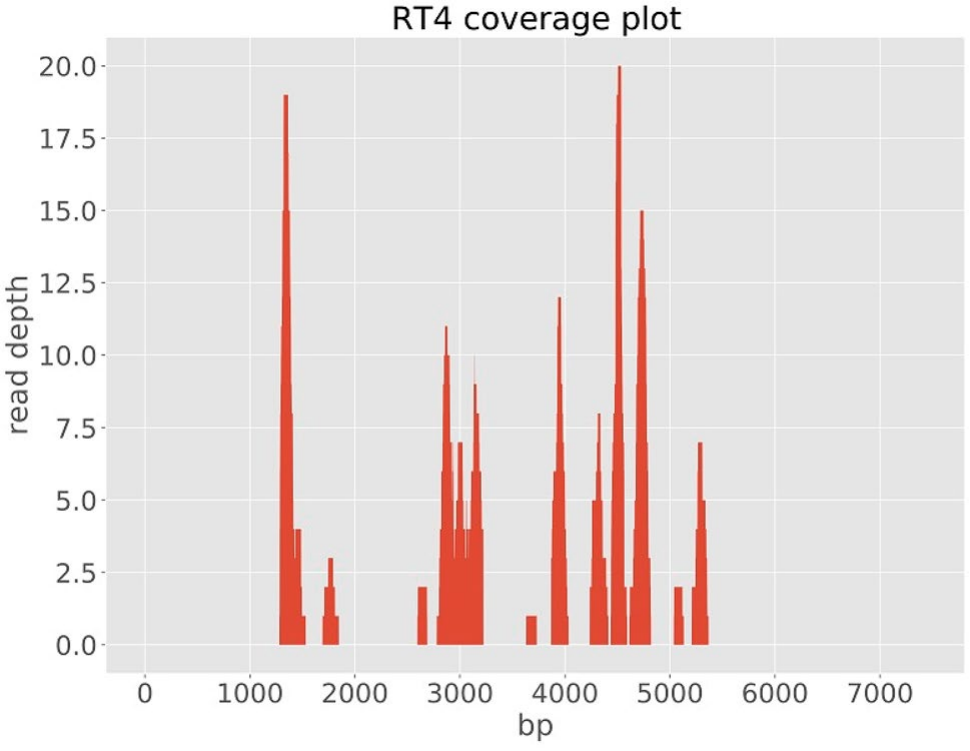
lllumina read coverage across Rabbit Hemorrhagic Disease Virus (GI genogroup) genome assembly from sample RT4*.*

**Figure 4:**
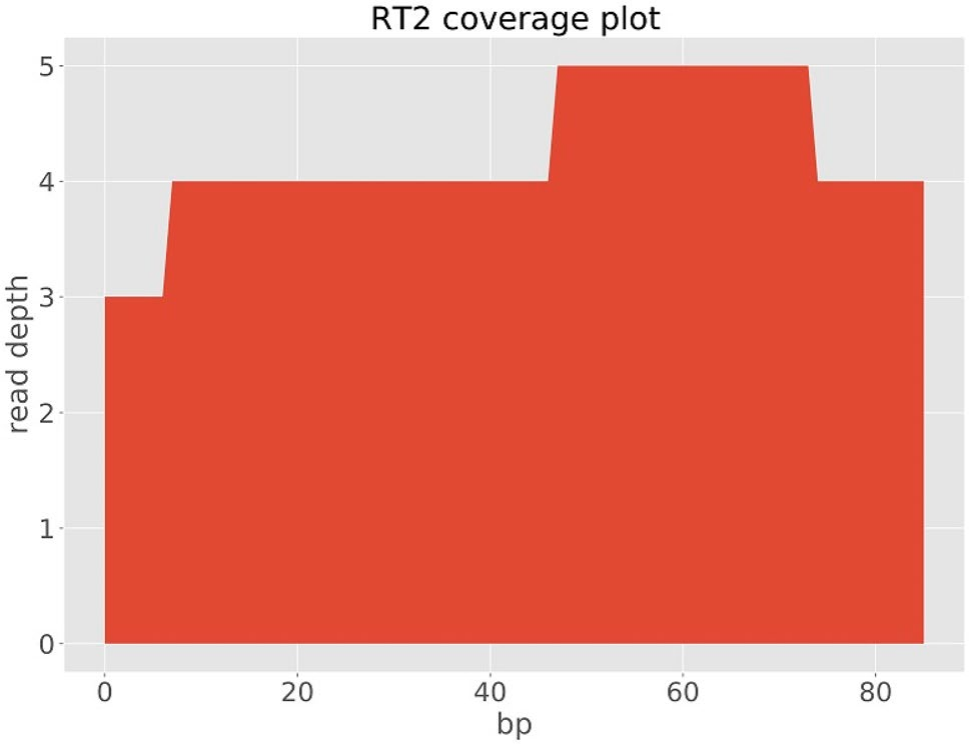
lllumina read coverage across Rabbit Hemorrhagic Disease Virus (GI genogroup) genome assembly from sample RT2*.*

We assembled three genomes; one full genome (sample RT5) and two partial sequences (samples RT4 and RT2), two of which (RT5 and RT4) were used for further analysis (Table 1). With the metagenomic analysis of the sequence data, genotype RHDV2 also known as GI.3P-GI.2 or RHDVb was identified in three samples (RT2, RT4 and RT5) out of five samples sequenced. The full genome (sample RT5) had a genome length of 6,976 bp and mean coverage depth of 16X. The two partial genomes had lengths of 1214 bp (sample RT4) and 86 bp (sample RT2) and mean coverage depths of 2X and 0.1X, respectively. Total number of reads for sample RT5 was 16,922 with a total number of RHDV2 reads of 1,455 (8.6% RHDV2 reads). Sample RT4 had 291,278 total reads and 1737 total RHDV2 reads (0.6% RHDV2 reads) while sample RT2 had 142,230 total reads and 126 total RHDV2 reads (0.09% RHDV2 reads). Mapping of our sequencing reads to the Rabbit Hemorrhagic Disease Virus (GI genogroup) reference genome showed that the reads mapped all across the genome of the virus. However, for samples RT4 and RT2 there wasn’t sufficient coverage (Figs. 2, 3, 4) to confidently call a base for most of the nucleotide positions and so for regions of ambiguity, our assembly pipeline called an ‘N’. Stripping RT2 and RT4 of all ‘Ns’ resulted in 86 bp and 1214 bp sequence lengths for each sample respectively.

**Figure 5:**
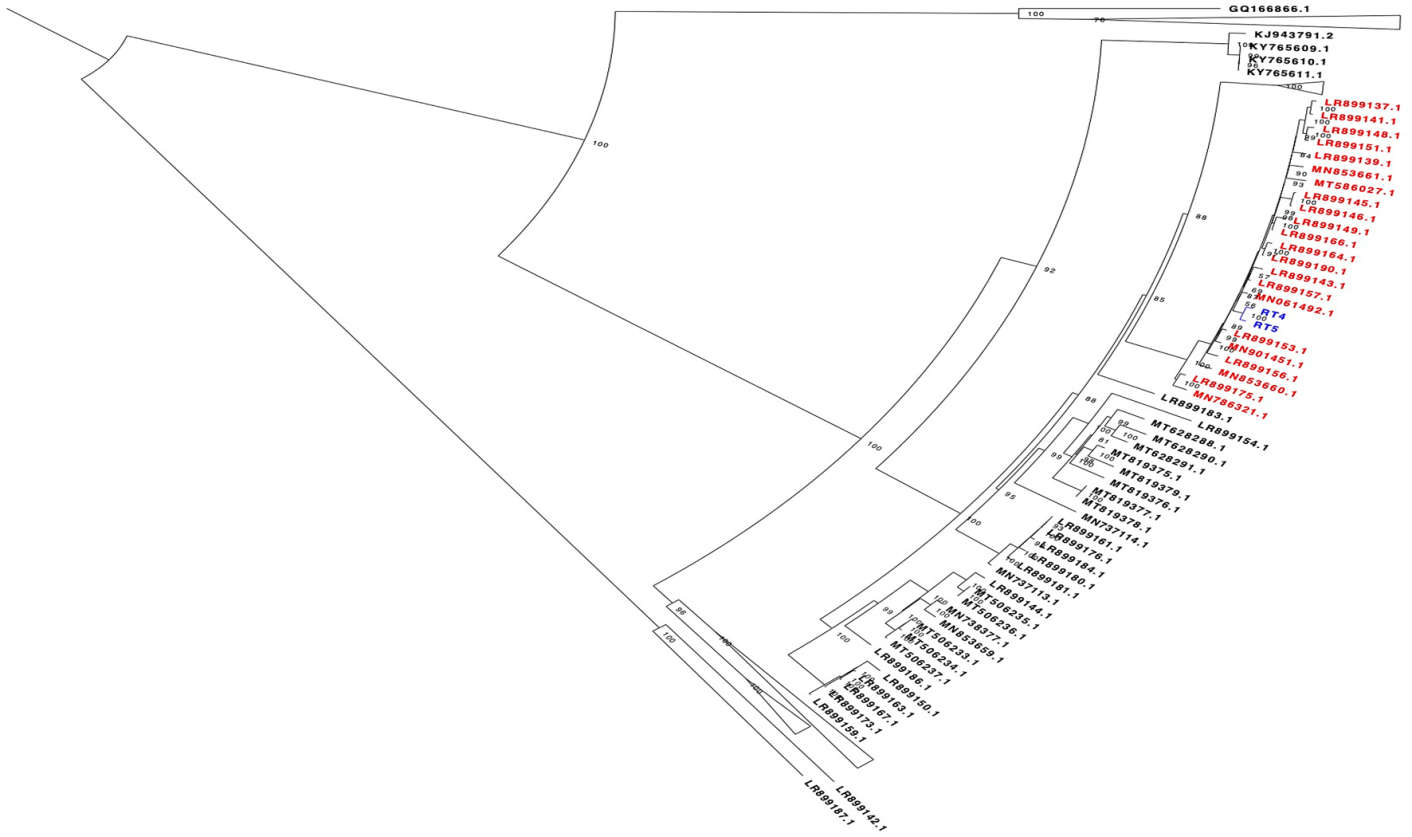
Mid-point rooted maximum likelihood phylogenetic tree showing relationship between the sequences from this study (coloured blue) and RHDV sequences obtained from the NCBI database. Sequences coloured red are sequences that are in the same clade as our study sequences and they are obtained from Germany, France, Netherlands, China and Poland. Bootstrap values are shown on the nodes**.**

BLAST analysis revealed that sample RT5 shared a 98.6% nucleotide pairwise identity with a 2016 Germany GI.2 sequence with NCBI accession number LR899157 and a 98.58% nucleotide pairwise identity with a 2016 Netherlands GI.2 sequence with accession number MN061492.1 across a 6,976 bp region. RT4 shared a 99.05% nucleotide pairwise identity with a 2016 Germany GI.2 sequence with NCBI accession number LR899157 across a 1214 bp region (Fig. 5). Phylogenetic analysis further confirmed the results of our BLAST analysis, demonstrating that the sequences from these animals belong to the GI.2 genotype as they clustered together in the same clade with previous GI.2 sequences from Europe and other parts of Africa (Fig. 5). Analysis of Single Nucleotide variants of sample RT5 revealed 11 common mutations resulting in amino acid changes. Four of these mutations (Leu1929Pro, Ser1978Phe, Val2104Ala, Val2127Ala) occur in the region of target for the RT-PCR1 primers.

## Discussion

Our initial suspicion of RHD based on case history, the extent of morbidity, mortality and postmortem findings was confirmed by RT-PCR investigation and metagenomic sequence analysis resulting in three GI.2 genome (one full and two partial) assemblies. In addition to metagenomic analysis, RDHV2 specific RT-PCR investigation showed all five samples to be positive.

To the best of our knowledge, this study provides the evidence of the first full genome sequence of Rabbit Hemorrhagic Disease Virus 2 (RHDV2) in sub-Saharan Africa.

The post mortem findings from this study were mild and not apparent in some of the carcasses. However, the few lesions observed were similar to some of the lesions reported in Europe^35^, Asia^36^, and Africa^39^. These suggest that the outbreak on the two farms were characterised by two clinical forms (acute and per acute form) of the disease.

Bioinformatics analysis of the virus genomes revealed that two sequences from this study belong to genotype *Lagovirus* europaeus/GI.2. This genotype was first identified in 2010 as a novel pathogenic form of *Lagovirus* in France^12^ after which it spread rapidly through Europe and other parts of the world (Oceania, North America, Asia and Africa)^22,23,29,30,32,33^. Lagovirus GI.2 has also been reported to replace the former circulating GI.1 genogroup in Australia and Portugal^37-39^. Phylogenetic analysis showed that our sequences clustered closely with previous sequences from Europe. BLAST analysis revealed sample RT5 shared a 98.6% nucleotide pairwise identity with a 2016 Germany GI.2 sequence with NCBI accession number LR899157, and a 98.58% nucleotide pairwise identity with a 2016 Netherlands GI.2 sequence with NCBI accession number MN061492.1 across a 6976 bp region. RT4 shared a 99.05% nucleotide pairwise identity with a 2016 Germany GI.2 sequence with NCBI accession number LR899157 across a 1214 bp region, suggesting that the virus was most likely imported into the country from Europe. In addition, the age of the European ancestral virus (2016) also suggests that the GI.2 found in our study was either an older introduction that circulated for some time undetected in Nigeria, or a more recent introduction of an unsampled, likely European virus.

The lagovirus europaeus/GI.3P-GI.2 genotype is known to dominate in most regions where it’s found. RHDV2 has also been circulating in some African countries and more recently, there have been reports of GI.2 outbreaks in a few countries from North Africa (Tunisia, Egypt and Morocco)^39,40^.

To the best our knowledge, there is no GI.2 genome sequence data from Sub-Saharan Africa despite its devastating effect on rabbit farming. This is due to the limited resources and scanty infrastructural facilities in sub-Saharan African countries for molecular investigations, disease monitoring and surveillance. In Australia, GI.2 spread to all states and territories and rapidly became the dominant circulating genotype within 18 months of initial detection^38^. Active surveillance and sequencing should be considered in order to understand the spread, diversity, host-virus interaction and their impact on the susceptible populations and the rabbit farming industry in Nigeria and Africa. Furthermore, analysis of more samples is also needed in order to determine the time of introduction of the virus into Nigeria and how the disease varies through the country.

Our phylogenetic analysis revealed high genetic diversity of the GI.3P-GI.2 genotype. The diversity found in our study is a characteristic of this virus. RT-PCR1 analysis of the samples targeting a 1740 bp of the VP60 capsid protein gene revealed only two positive samples out of five. The reason why RT5 gave a negative result during RT-PCR1 and yet yielded a full genome after metagenomic analysis, could be as a result of accumulation of mutations, some of which occurred in the target region of the primers. This further emphasizes the genetic diversity, which is likely due in part to the rapid spread and evolution of this virus. This is also within the expectations for RNA viruses that have been circulating for more than 10 years now; this probably reflects lack of sampling and sequencing of GI.2 strains from Africa and the limited comparison with RHDV strains. Therefore, compiled genomic data should be carefully considered when developing diagnostics and updating already available vaccines.

The findings from this study is a significant landmark in the field, as it has revealed the circulation of GI.2 in Nigeria, and reports the first genomic characterization of RHDV2 in sub-Saharan Africa. The close sequence homology suggests that the virus was most likely imported from Europe. In addition, the high genetic diversity of the GI.2 genogroup found in our study highlights the need for characterization of many more samples across sub-Saharan Africa, in order to guide the development of improved diagnostics and update RHDV2 vaccines.

Furthermore, the need for unbiased metagenomic analysis for diagnosis of suspected cases and discovery of new variants couldn’t be overemphasized. In this study, only one full genome was assembled despite the RT-PCR positivity of all the samples tested to GI.2 virus. This may be due to the sequencing method used and or sample preparation for the mNGS. Sample quality in addition to the sequencing throughput method, has tremendous impact on the method sensitivity, particularly the ability to detect small amounts of virus in the background of host nucleic acids^41^. It is in a bid to enhance viral nucleic acid detection that many authors have described viral enrichment approaches for RNA virus discovery in clinical samples^42–44^. In this study also, no targeted RNA virus discovery strategies were applied on the tissue samples to enrich viral sequences. Perhaps, these enrichment steps if done, would have provided a more effective and in depth viral nucleic acid detection with more successful full genome assemblies.

Overall, the detection of RHDV2 with unbiased metagenomic sequencing, as shown in this study illustrates the power of genomics in explaining a suspected outbreak. This ability to rapidly identify and characterize an emerging virus (RHDV2) highlights the value of in-country genomics capacity. Serology using ELISA and RT-PCR are the current methods of choice for RHDV diagnosis in Sub-Saharan Africa. These diagnostic methods despite their limitations are done in very few selected laboratories. The integration of genomics capacity into the established, but siloed, pathogen-specific diagnostic platforms provides exciting opportunities for Veterinary public health surveillance.

## Methods

### Post mortem and sample collection

In August 2020, following reports of devastating outbreaks of suspected Rabbit haemorrhagic disease (RHD) in rabbitries in Ibadan, South-western region of Nigeria, post mortem was carried out on four carcasses from farm A and one carcass from farm B. Signalment from farm A consists of one female (8 weeks old) and three males (8 weeks, 5 weeks and 3 weeks old). Farm B consisted of one adult male rabbit. The breeds on both farms were Hyla. Tissue sections were collected into RNAlater from the five (5) rabbit carcasses suspected to have died from RHD. The samples were tagged RT1, RT2, RT3, RT4 (farm A) and RT5 (farm B). Tissues (liver, spleen, lungs) of each animal were pooled for RT1-RT4, while only liver was collected for RT5. The samples were then maintained in a cold chain and RNALater during transportation to the African Centre of Excellence for the Genomics of Infectious Disease (ACEGID), Redeemer’s University, Ede, Nigeria for PCR confirmation and metagenomics sequencing analysis.

### RNA extraction

Samples stored in RNAlater were first washed in PBS and thereafter homogenized and macerated. Total RNA was extracted from tissues macerated in TRIzol using QIAamp Viral RNA extraction kit (Qiagen, Hilden, Germany) according to manufacturer’s instructions. Extracted RNA was stored in − 20 °C until RT-PCR and sequencing.

### Next generation sequencing and bioinformatics analysis

Upon RT-PCR confirmation on September 25, 2020, Nextera XT sequencing Libraries were made based on established unbiased protocol^45,46^ as routinely used in our laboratory. Briefly, host ribosomal RNA were quantified by rt-qPCR and samples with over a million copies per microlitre depleted using rRNA probes. Extracted RNAs were cleaned from unwanted nucleic acid using Turbo DNase treatment and converted to cDNA by a random primer hybridization. Subsequently, Nextera XT sequencing libraries were made and quantified by KAPA qPCR.

Normalized and pooled libraries were quantified via KAPA qPCR and the fragments size were determined using BioAnalyser. The pool was loaded on the Miseq at a final concentration of 10 pM. Using the Illumina Miseq V2 -300 cycle cartridge with read length 101 and 2-channel SBS chemistry, we carried out a paired-end sequencing to ensure high quality reads from both ends of the sequencing library.

Following sequencing, raw reads from the next-generation sequencing machine were uploaded to our cloud-based platform (DNAnexus, www.dnanexus.com). Quality control was carried out on the raw reads using fastqc (https://www.bioinformatics.babraham.ac.uk/projects/fastqc). Metagenomics analysis was carried out using Kraken2^4^.

RHDV genomes were assembled using our publicly-available software viral-ngs v2.1.8 (https://github.com/broadinstitute/viral-ngs) implemented on DNAnexus. Following BLASTn analysis, all whole genome RHDV sequences available in GenBank as at 24th March 2021 were aligned with two of our sequences using MAFFT v7.453^47^ Using Geneious Prime 2021.0.3^48^. A 4127 bp region was identified as having the most coverage by all the sequences; this region was extracted and used to infer a maximum likelihood tree using IQTREE v1.6.12^49^. IQtree ModelFinder^50^ selected SYM + R5 as the best-fit model according to Bayesian Information Criterion (BIC) for the dataset and ultrafast bootstrap^51^ with 1000 replicates was carried out. The tree was viewed and manually edited using FigTree (http://tree.bio.ed.ac.uk/software/figtree/). The two sequences from this study were aligned with all whole genome sequences obtained from NCBI to check for amino acid mutations specific to our new sequences from Nigeria. We also mapped the RT-PCR primers to our full genome obtained from this study to check for any mutations in the target regions of the primers.

### RT-PCR

We conducted two different sets of RT-PCRs (RT-PCR1 and RT-PCR2 for the purpose of this study) on extracted RNA with modified established protocols^16^. For RT-PCR1, primers (RHD-F5′-ATGGAGGGCA AAGCCCGCACAGCG-3′ and RHD-R 5′- AATTCAGACATAAGAAAAGCCA TTG-3′) targeting the VP60 capsid protein gene giving a 1740 base pair product were used. One-step SuperScript III One-Step RT-PCR System with Platinum Taq DNA Polymerase (Invitrogen, USA) was used for PCR amplification. Following positive metagenomics sequencing data from some negative samples in RT-PCR1, a second RT-PCR (RT-PCR2) using a different set of primers was conducted to confirm the presence of the lagovirus in all five samples collected from RHD suspected animals. We used RHDV specific primers; forward 5′-GTT ACG ACT GTG CAG GCC TAT GAG TT-3′ and reverse 5′-TTG TTG AGC AGT CCA ATT GTC ACT G-3′ in this experiment to target a 398-bp region of the VP60 (capsid) gene^52^**.**

Both RT-PCRs final reaction volume of 25ul were made up of 12.5 µl of 2X reaction mix, 1.25 µl of 20 µM each of forward and reverse primers, 1 µl SuperScript III RT/Platinum Taq Mix, 1 µl RNA template and MgSo4 optimization to a final concentration of 2.5 µM and nuclease free water to make up the reaction volume. The cycling conditions RT-PCR1 included; 55 °C for 30 min for cDNA synthesis, pre-denaturation at 95 °C for 15 min, then 40 cycles at 95 °C for 1 min, 58 °C for 30 s and 72 °C for 1 min, and a final extension step at 72 °C for 10 min. While the cycling conditions for RT-PCR2 were as follows: cDNA synthesis at 50 °C for 30 min; then pre-denaturation at 95 °C for 15 min and 43 cycles of 94 °C for 30 s, 55 °C for 30 s, and 72 °C for 1 min; and final extension of 72 °C for 10 min. RT-PCR products were viewed in 1% and 2% gel electrophoresis for RT-PCR1 and RT-PCR2, respectively.

The samples were all confirmed positive for a lagovirus and this was confirmed to be RHDV2 by sequencing and bioinformatic analysis.

## Data Availability

All sequences from this study were submitted to the National Center for Biotechnology Information (NCBI) database/GenBank and the accession numbers (MW123059–MW123061) received on the 16 October, 2020.
